# Suppressors of Meiotic Silencing by Unpaired DNA

**DOI:** 10.3390/ncrna5010014

**Published:** 2019-01-28

**Authors:** Hua Xiao, Thomas M. Hammond, Patrick K. T. Shiu

**Affiliations:** 1 Division of Biological Sciences, University of Missouri, Columbia, MO 65211, USA; xiaohu@missouri.edu; 2 School of Biological Sciences, Illinois State University, Normal, IL 61790, USA

**Keywords:** meiosis, meiotic silencing by unpaired DNA (MSUD), *Neurospora crassa*, RNA interference (RNAi), small interfering RNA (siRNA)

## Abstract

Meiotic silencing by unpaired DNA (MSUD) is a gene silencing process that occurs within meiotic cells of *Neurospora crassa* and other fungi. We have previously developed a high-throughput screen to identify suppressors of this silencing pathway. Here, a list of MSUD suppressor candidates from a single pass of the first 84 plates of the *Neurospora* knockout library is provided.

## 1. Introduction

Meiosis in *Neurospora crassa* begins with the fusion of two haploid nuclei, one from each parent of a sexual cross. After the nuclear fusion, the seven chromosomes from each parent are grouped into pairs of homologs and scanned for unpaired intervals of DNA. Any gene that does not have a proper pairing partner is silenced for the duration of sexual development within the ascus (spore sac). The mechanism that detects and silences unpaired genes is known as meiotic silencing by unpaired DNA (MSUD) [1]. MSUD plays a key role in genome surveillance and is capable of targeting selfish genetic elements during the sexual stage [2].

Several proteins involved in MSUD, including common RNA interference (RNAi) factors, have been characterized [3]. Silencing-deficient strains that were first identified through an MSUD genetic screen are known as *suppressor of ascus dominance* (*sad*) mutants [1]. The reported *sad* mutations were named as such because they have the ability to suppress certain “ascus-dominant” mutations, some of which were later found to be dominant over wild type (WT) due to a deletion (thus depriving the WT gene a pairing partner). Silencing genes that were previously described for an unrelated function in *Neurospora* and/or discovered through sequence similarity are not usually given the *sad* prefix. A working MSUD model begins with the detection of an unpaired DNA region, possibly with the help of the SAD-6 homology search protein [4]. A single-stranded aberrant RNA (aRNA) is produced and transferred to the perinuclear region, where the meiotic silencing complex (MSC) examines any exported RNAs [5]. One of the MSC components, the SAD-1 RNA-directed RNA polymerase (RdRP), converts the aRNA into a double-stranded RNA (dsRNA) [6]. SAD-3, an RNA helicase, may assist SAD-1 by increasing its processivity on RNA templates [7]. The dsRNA is then cut into small interfering RNAs (siRNAs) by the DCL-1 Dicer [8]. With the assistance of the QIP exonuclease, the siRNAs are turned into single strands, which subsequently guide the SMS-2 Argonaute to target complementary mRNAs for silencing [9,10]. The SAD-2 scaffold protein functions to bring SAD-1 and others to the perinuclear region [5,11]. SAD-4, SAD-5, and SAD-7 have unclear roles in silencing, although it is known that the first two factors are necessary for siRNA production and the last one may coordinate nuclear and extranuclear events [12,13,14]. Finally, CBP20 and CBP80 form the cap-binding complex (CBC), which helps transport mRNAs to the perinuclear MSC [15].

The first two *sad* genes were identified through forward genetic screens and functional cloning, which are quite time-consuming [6,11]. In 2011, a high-throughput screening procedure for the identification of MSUD mutants was published, along with the characterization of a single suppressor (*sad-3*^Δ^) identified from the screen [7]. All subsequently reported *sad* mutations were identified using this method, which involves a systematic examination of single deletion mutants in the *Neurospora* knockout library [16]. Here, we present a list of candidate *sad* suppressors in hopes that it may expedite the pace of gene silencing research.

## 2. Results and Discussion

### 2.1. Identification of Candidate MSUD Genes

A *Neurospora* cross typically produces ascospores (sexual spores) that are shaped like an American football and black in color. Genetically, the shape and color of an ascospore depend on the *Round spore* (*r*^+^) and *Ascospore maturation-1* (*asm-1*^+^) loci, respectively. Accordingly, if *r*^+^ were meiotically unpaired (i.e., *r*^+^ × *r*^∆^), it would be silenced during sexual development, resulting in the production of mainly round spores [1]. Similarly, if *asm-1*^+^ were unpaired, predominantly white (unmelanized) spores would be generated. These atypical phenotypes can be alleviated if silencing is defective, such as in a cross where an MSUD gene is itself unpaired (e.g., *sad*^+^ × *sad*^∆^) and thereby silenced through a negative feedback loop. This self-silencing scheme is known as “silencing the silencer” [3], and it provides a method to determine if a gene is involved in MSUD. Specifically, by testing each knockout strain’s ability to suppress the silencing of unpaired *r*^+^ and *asm-1*^+^, we can isolate mutant candidates that are deficient in MSUD.

As seen in Table 1, screening of the first 84 plates of the *Neurospora* knockout library revealed 24 candidate MSUD suppressors. Five of these candidates, also known as *sad-3* to *sad-7*, have been published and discussed above. Other candidate genes encode a wide range of proteins, such as an Argonaute-binding protein, protein kinases, RNA-binding proteins, and other proteins with various functional domains. While it is easy to explain how an Argonaute-binding protein could be involved in gene silencing (e.g., via siRNA loading) [17,18], the roles of other SAD protein candidates would require extensive studies to decipher.

Although an entry in Table 1 can demonstrate how well a *sad* mutant candidate acts as a semidominant suppressor of a particular silencing phenotype, it does not indicate whether the corresponding gene is critical for MSUD. For example, a *sad-5*^∆^ × *r*^∆^ cross produces only 0.4% football progeny, giving an impression that *sad-5* may be nonessential to silencing. However, an *r*-unpaired cross homozygous for the *sad-5* deletion produces almost 100% football progeny, suggesting that this *sad* gene is absolutely required for MSUD [13]. Conceivably, the degree of semidominant suppression in our assay may be a reflection of the expression timing/level of both the *sad* gene and the unpaired tester gene, i.e., the *r*^∆^ tester may be better at detecting certain candidates, while the *asm-1*^∆^ tester may be better for the others. Accordingly, at least two different testers should be used for this type of screening in order to minimize the potential for missing genuine suppressors.

### 2.2. Future Directions

MSUD is known to be present in two *Neurospora* and one *Fusarium* species [1,21,22]. Examination of the aforementioned *sad* candidates in these fungi should shed light on the molecular pathway starting from unpaired DNA detection to silencing of homologous transcripts. The knockout library plates (1–84) analyzed in this study contain deletion mutants of over five thousand loci. Considering that the *Neurospora* genome consists of only about 10 thousand protein-coding genes [23], our screening here represents a good start towards the genetic dissection of MSUD. Since cross-contamination and questionable authenticity are known issues of the arrayed knockout plates (http://www.fgsc.net/NeurosporaGenomeProject/arrayedkoplates.htm), future work should include a comprehensive investigation of the remaining 19 candidates to confirm that the documented mutations are indeed responsible for the suppression phenotypes. For a thorough testing of the knockout library, it is a good idea to rescreen the first 84 plates (in case candidates are missed due to poor mating) as well as inspect the remaining ones (i.e., plates 85–136).

## 3. Materials and Methods

### 3.1. Strains, Media, and Fungal Manipulation

Tester and control strains used in this study are listed in Table 2. The *Neurospora* knockout library was obtained from the Fungal Genetics Stock Center (FGSC) [24]. Vogel’s medium and synthetic crossing (SC) medium were used for vegetative growth and sexual crosses, respectively [25,26]. Standard techniques from the *Neurospora* protocol guide were employed throughout this study (http://www.fgsc.net/Neurospora/NeurosporaProtocolGuide.htm).

### 3.2. MSUD Assays

A high-throughput screen for MSUD mutants has been described previously [7]. In brief, female testers grown within the wells of a library plate (Corning 3894) were fertilized by replicator-transferred conidia, and shot ascospores were evaluated directly on the lid using a dissecting microscope. Candidate MSUD suppressors were re-examined using a low-throughput assay performed on individual 60 mm petri dishes (Fisher FB0875713A) [7] or as follows: female testers were cultured in 24-well microplates (Corning 3524) containing SC medium for 6–7 days. Each tester strain within a well was fertilized by 50 µL conidial suspension (1000 counts/µL) of a candidate MSUD suppressor. Two to three replicate crosses were performed for each combination of tester (female) and candidate suppressor (male). After fertilization, the microplates were incubated in standard 1020 greenhouse trays (with clear humidity domes) at room temperature and under ambient lighting for 21–24 days. Shot ascospores were collected from the undersides of microplate lids, suspended in water, and examined for spore shape and/or color under magnification. With the exception of *sad-1* (which was used as a control), known MSUD mutants identified in the initial screen were not subjected to a low-throughput assay. 

## Figures and Tables

**Table 1:** List of *sad* mutant candidates and their semidominant suppression of MSUD.

Strain	Gene No.	*r*^∆^ (% Football)	*asm-1*^∆^ (% Black)	Predicted Product *
**Candidate**				
*a* (*sad-4*) *	*ncu01591*	52.4 ± 3.9	61.6 ± 2.5	Hypothetical protein
*b* (*sad-7*) *	*ncu01917*	53.2 ± 6.5	67.3 ± 4.3	RNA-binding protein (RRM)
*c*	*ncu00563*	0.0 ± 0.0	25.4 ± 6.6	Argonaute binding protein-2
*d*	*ncu04326*	56.2 ± 12.5	11.0 ± 5.6	Serine/threonine protein kinase
*e*	*ncu03578*	82.1 ± 11.1	10.6 ± 11.7	SbcC-domain protein
*f*	*ncu00095*	44.3 ± 6.0	3.6 ± 0.8	RNA polymerase-associated protein (Ctr9)
*g*	*ncu02068*	28.3 ± 16.5	11.2 ± 6.5	BRCT-domain protein
*h*	*ncu16560*	15.7 ± 7.3	28.2 ± 13.1	RNA-binding protein (Pumilio)
*i*	*ncu02964*	4.2 ± 1.0	27.8 ± 3.6	Hypothetical protein
*j*	*ncu02152*	2.1 ± 0.4	25.6 ± 12.4	RNA-binding protein (RRM)
*k*	*ncu03174*	2.3 ± 1.9	10.7 ± 3.7	Hypothetical protein
*l*	*ncu04067*	15.9 ± 0.6	10.5 ± 3.0	Tht1-like nuclear fusion protein
*m*	*ncu03483*	26.8 ± 4.3	10.9 ± 1.2	Dynactin-1
*n*	*ncu03715*	0.2 ± 0.3	32.7 ± 15.8	Kinesin-3
*o* (*sad-3*) *	*ncu09211*	81.2 ± 7.8	28.2 ± 9.0	RNA helicase
*p* (*sad-6*) *	*ncu06190*	40.4 ± 15.3	40.0 ± 6.6	SNF2-family protein
*q* (*sad-5*) *	*ncu06147*	0.4 ± 0.1	15.9 ± 0.9	Hypothetical protein
*r*	*ncu06882*	28.0 ± 2.5	35.4 ± 2.4	Ubiquitin-protein ligase
*s*	*ncu07872*	2.1 ± 1.4	14.9 ± 2.5	Dual-specificity protein kinase (Yak1)
*t*	*ncu09064*	14.6 ± 0.5	28.7 ± 13.1	Serine/threonine protein kinase
*u*	*ncu06213*	2.0 ± 1.0	6.9 ± 0.1	Zinc finger transcription factor (MIZ)
*v*	*ncu06461*	9.4 ± 3.3	2.6 ± 0.9	Pentatricopeptide repeat protein
*w*	*ncu00331*	4.1 ± 1.1	47.8 ± 4.8	Hypothetical protein
*x*	*ncu04104*	2.3 ± 1.0	27.3 ± 1.2	Chromosome segregation protein (Cse1)
**Control**				
*sad-1*	*ncu02178*	98.7 ± 1.0	84.8 ± 0.8	RNA-directed RNA polymerase
WT		0.0 ± 0.0	0.7 ± 0.1	

* The information is from FungiDB [19], NCBI’s conserved domain database [20], the *Neurospora* e-Compendium (http://www.bioinf.leeds.ac.uk/~gen6ar/newgenelist/genes/gene_list.htm), and/or a previous *sad* paper [1,4,7,13,14].

**Table 2:** Tester and control strains used in this study.

Strain	Genotype
F2-25	*rid his-3* ^+^ *::asm-1^+^; fl; asm-1* ^∆^ *::mtr^+^ A*
F2-27	*rid r* ^∆^ *::hph; fl; a*
F2-29	*rid r* ^∆^ *::hph; fl; A*
F3-02	*his-3*^+^*::asm-1^+^; fl; asm-1*^∆^*::mtr^+^ a* heterokaryon with *fl; nic-3;* ∆*mat*
F3-23	*rid his-3* ^+^ *::asm-1^+^; fl; asm-1* ^∆^ *::hph A*
F3-24	*rid his-3* ^+^ *::asm-1^+^; fl; asm-1* ^∆^ *::hph a*
P3-07	Oak Ridge wild type (WT) *A* (FGSC 2489)
P3-08	Oak Ridge wild type (WT) *a* (FGSC 2490)
P3-25	*mep sad-1* ^∆^ *::hph a*
P8-18	*mep sad-1* ^∆^ *::hph A*

Genetic loci are described in the *Neurospora* e-Compendium.
